# An Elevated Peripheral Blood Monocyte-to-Lymphocyte Ratio Predicts Poor Prognosis in Patients with Primary Pulmonary Lymphoepithelioma-Like Carcinoma

**DOI:** 10.1371/journal.pone.0126269

**Published:** 2015-05-07

**Authors:** Liang Wang, Wen Long, Peng-fei Li, Yong-bin Lin, Ying Liang

**Affiliations:** 1 State Key Laboratory of Oncology in South China; Collaborative Innovation Center for Cancer Medicine, Guangzhou, Guangdong, People’s Republic of China; 2 Department of Hematologic Oncology, Sun Yat-sen University Cancer Center, Guangzhou, Guangdong, People’s Republic of China; 3 Department of Nuclear Medicine, Sun Yat-sen University Cancer Center, Guangzhou, Guangdong, People’s Republic of China; 4 Department of Pediatric Oncology, Sun Yat-sen University Cancer Center, Guangzhou, Guangdong, People’s Republic of China; 5 Department of Thoracic Surgery, Cancer Center, Sun Yat-sen University, Guangzhou, Guangdong, People’s Republic of China; 6 Department of Medical Oncology, Cancer Center, Sun Yat-sen University, Guangzhou, Guangdong, People’s Republic of China; Peking University People Hospital, CHINA

## Abstract

Primary pulmonary lymphoepithelioma-like carcinoma (LELC) is a rare type of non-small cell lung cancer. In this study, we retrospectively reviewed the data from 74 consecutive patients with pulmonary LELC and investigated the prognostic value of pretreatment monocyte-to-lymphocyte ratio (MLR). The cut-off value determined by ROC curve for MLR was 0.262. According to this cut-off value, 36 (48.6%) patients had lower MLR value (<0.262) at diagnosis. There was no significant correlation between MLR level and gender, age, smoking history, stage, and lactate dehydrogenase (LDH) level. The 2-year, 5-year, and 10-year OS rate were 86%, 72%, and 61%, respectively; the 2-year, 5-year, and 10-year PFS rate were 71%, 63%, and 49%, respectively. In univariate analysis, advanced stage, elevated LDH level, and higher MLR value (> = 0.262) were significantly associated with poor OS and PFS. In a multivariate Cox regression model that included stage, LDH and MLR level, all of these three factors were found to be independent prognostic factors for both PFS and OS. In patients who received radical surgery, MLR level remained significantly correlated with OS and PFS. In conclusion, we firstly demonstrated that pretreatment MLR can be used as a useful independent prognostic marker in patients with pulmonary LELC, and might guide us to optimize the treatment strategies. However, due to the relatively rarity of this disease and the limitation of a retrospective study, further prospective studies performed in multicenter are necessary to validate the prognostic value of MLR in pulmonary LELC.

## Introduction

Primary pulmonary lymphoepithelioma-like carcinoma (LELC), a rare type of non-small cell lung cancer (NSCLC), was firstly reported by Begin et al [1] in 1987, and is categorized as a subtype of large cell carcinoma [2]. It is reported to be closely related to the infection of Epstein-Barr virus (EBV), and has similar histology to nasopharyngeal carcinoma [3], which is a type of undifferentiated carcinoma with predominant lymphocytic infiltration. Recent studies have found that patients with pulmonary LELC have significantly better prognosis than other types of NSCLC [4]. However, due to the rarity of this disease, treatment strategy is still controversial. Radical surgery is usually performed for early stage disease, and combination of chemotherapy and radiotherapy has been used in locally advanced or metastatic disease. In recent years, increasing case reports or cohort studies have been published, mainly focusing on the treatment approaches or exploration of driving genes, butno large cohort studies have ever been reported to investigate prognostic factors for pulmonary LELC. Thus, it is of great importance to find some valuable prognostic factors to guide treatments.

It is now widely recognized that cancer-associated inflammation is very common in tumor progression and associated with survival in variety of cancers [5]. Lymphocytes and monocytes are the main type of immune cells involved in the inflammatory process and have been demonstrated to be associated with the prognosis of many malignancies [6–9]. Recently, the pretreatment lymphocyte-to-monocyte ratio (LMR) or monocyte-to-lymphocyte ratio (MLR) has been shown to be a prognostic factor for clinical outcomes in diffuse large B-cell lymphoma[8] and lung cancers[10]. Therefore, we hypothesized that MLR may also play an important role in pulmonary LELC. We performed a large retrospective cohort study on patients with pulmonary LELC and investigated the prognostic value of pretreatment MLR. To our knowledge, this is the first large-scale study on the association between MLR and LELC.

## Materials and Methods

### Patients

74 consecutive patients with pulmonary LELC from January 2001 to December 2013 were enrolled in this study. As previously reported [4], patients with negative Epstein-Barr virus (EBV)-encoded RNA (EBER) staining were excluded in this study. Nasopharyngoscopy or PET-CT was done to rule out lung metastasis from nasopharyngeal carcinoma. Sun Yat-sen University Cancer Center Research Ethics Board has approved us to use the data in this study, and written informed consent for use and publication of patients’ medical information was obtained from all patients at their first visit.

We restaged all cases based on the American Joint Committee on Cancer (AJCC) staging system (the 2007 TNM Classification of Malignant Tumors)[11], and both clinical and pathological characteristics were reviewed. The MLR was calculated based on the whole blood cell counts at diagnosis. Treatment response was evaluated after at least 2 cycles of chemotherapy.

### Statistical analysis

Receiver operating characteristic (ROC) curve was performed to search the best cut-off value for MLR to stratify patients at a high risk of death (using SPSS version 19 statistical software). In this ROC curve, the point with the maximum sensitivity and specificity was selected as the best cut-off value. Progression-free survival (PFS) and overall survival (OS) were calculated by the Kaplan–Meier method, while log-rank test was used for comparison. OS was calculated from the date of diagnosis to the date of death from any cause, and was censored at the date of last follow-up interview. PFS was calculated from the date of diagnosis to the date of disease progression, relapse, or death from any causes, whichever came first. The prognostic factors of OS and PFS were analyzed by univariate analysis. Multivariate analysis was performed using the Cox regression model to compare the factors proven significant in the univariate analysis. Relative risk and 95% confidence interval were calculated for all variables in the regression model. A two-sided p-value < 0.05 was considered statistically significant. SPSS version 19 statistical software was utilized.

## Results

### Clinical characteristic of pulmonary LELC patients

The clinical characteristics are demonstrated in Table 1. Thirty-five (47.3%) patients were male, and the median age at diagnosis was 50 years (range, 9–74 years). Only 16 (21.6%) patients were smokers. The stages at initial diagnosis were: 27 (32.1%) in stage I, 15 (20.3%) in stage II, 27 (32.1%) in stage III, and 5 (6.8%) in stage IV. In this cohort, 28 patients (37.8%) received radical surgery alone, 36 patients (48.7%) received multimodality treatment strategy (radical surgery combined with radiotherapy or chemotherapy), and 10 patients (13.5%) received palliative chemotherapy.

**Table 1:** Clinical characteristics of 74 patients with pulmonary LELC.

Parameters		Number of patients (N = 74)	Percentage (%)
Gender	Male	35	47.3
	Female	39	52.7
Age	<60	60	81.1
	> = 60	14	18.9
Smoking history	No	58	78.4
	Yes	16	21.6
Stage	I-II	42	56.8
	III-IV	32	43.2
Hemoglobin level	<120g/L	13	17.6
	≥120g/L	61	82.4
Serum LDH level	Normal	62	83.8
	Elevated	12	16.2
Serum albumin level	<40g/L	37	50.0
	≥40g/L	37	50.0
Tumor size	≤3 cm	25	33.8
	>3 cm	49	66.2
MLR*	<0.262	36	48.6
	> = 0.262	38	51.4
Treatment	Radical surgery alone	28	37.8
	Combination of surgery and chemotherapy or radiotherapy	36	48.7
	Chemotherapy alone	10	13.5
Outcome	Died	20	27.0
	Alive	54	73.0

*the cutoff value of MLR was calculated based on the analysis of AUC curve.

### Determination of the optimal cut-off value of MLR

**Fig 1:**
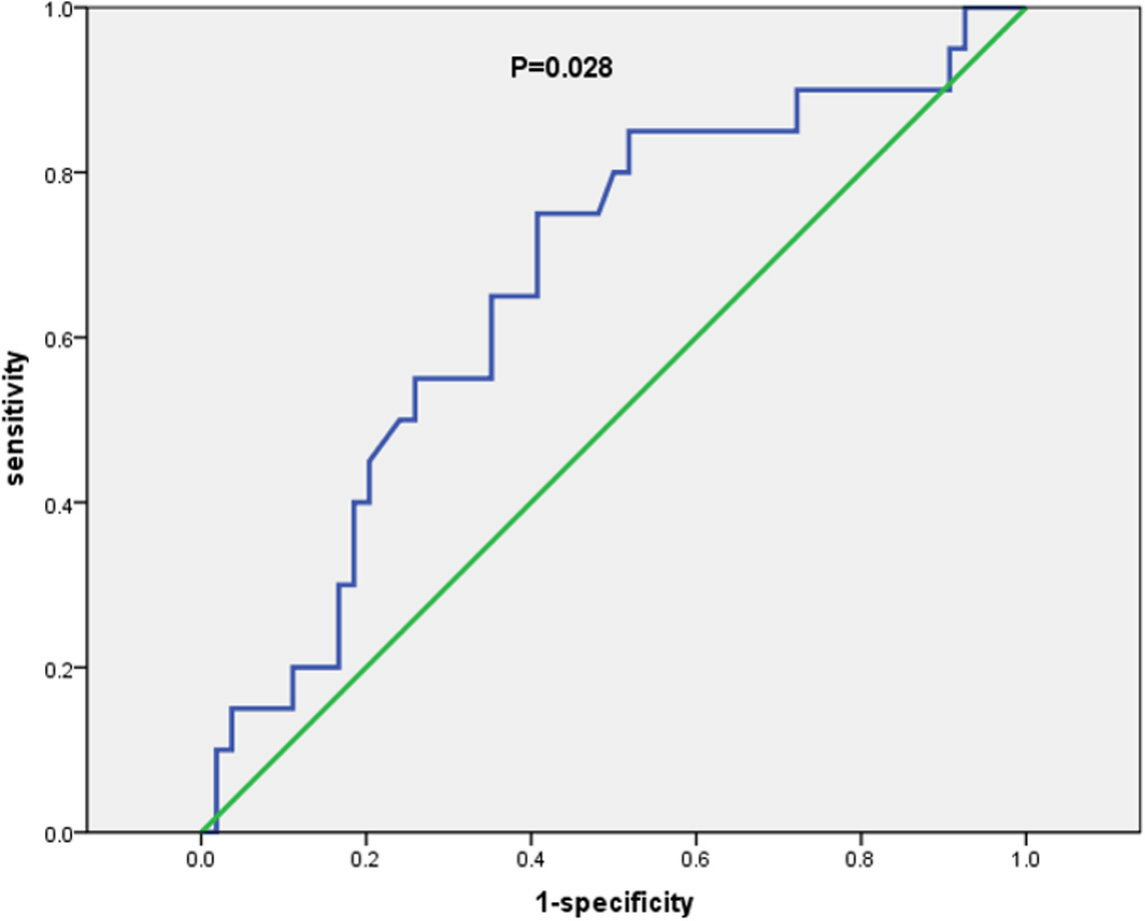
ROC curve analysis for the optimal cut-off point of MLR. The most discriminative cut-off value of MLR was 0.262 with an AUC value of 0.667 (p = 0.028). The sensitivity and specificity were 75.0% and 60.0%, respectively.

The median value of the pretreatment lymphocyte was 1.80×10^9^/L (range 0.90–3.70×10^9^/L), and the median value of pretreatment monocyte was 0.50×10^9^/L (range 0.10–1.40×10^9^/L). The optimal cut-off value of MLR was defined to be 0.262 by ROC curve with an AUC of 0.667 (p = 0.028) **(Fig 1)**. According to this cut-off value of MLR, 36 (48.6%) patients had lower MLR value (<0.262) at diagnosis. There was no significant correlation between MLR level and gender, age, smoking history, stage, and LDH level (P>0.05).

### Survival analysis

**Fig 2:**
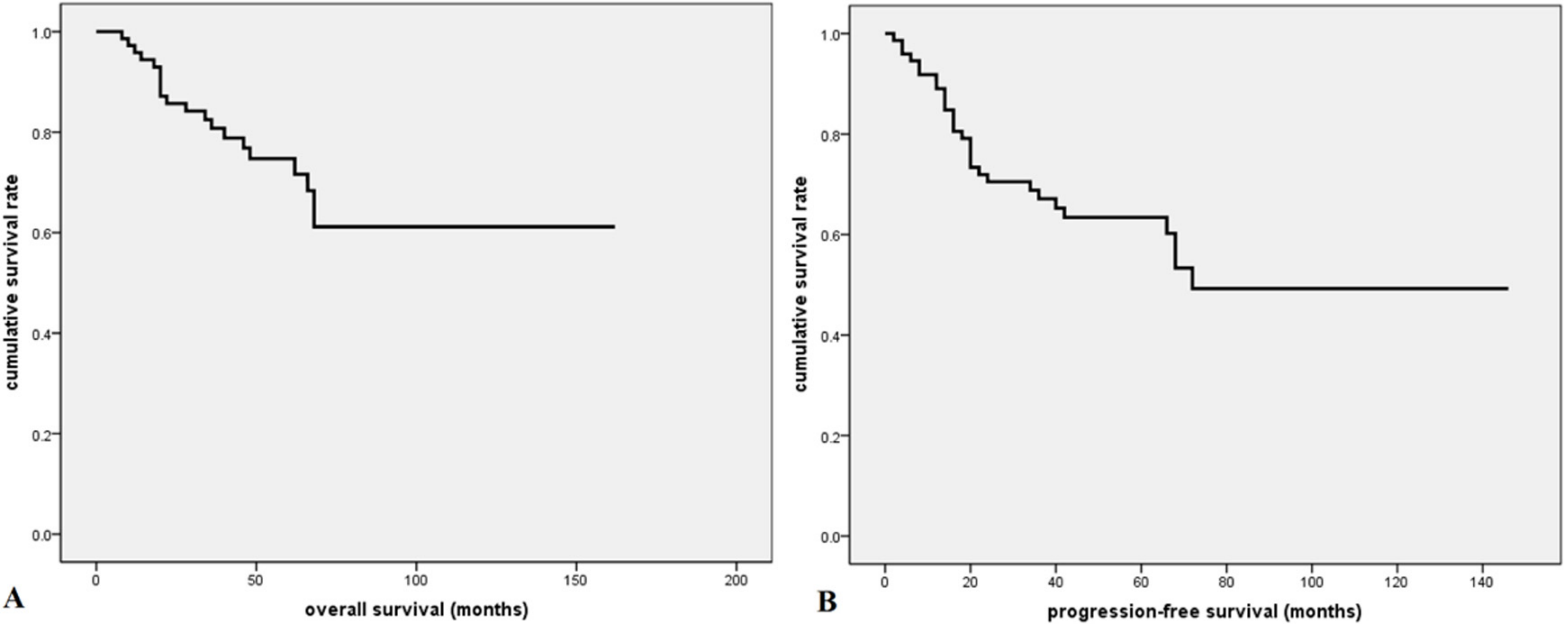
Survival curves for all patients with pulmonary LELC. A. The 2-year, 5-year, and 10-year OS rate were 86%, 72%, and 61%, respectively; B. The 2-year, 5-year, and 10-year PFS rate were 71%, 63%, and 49%, respectively.

**Fig 3:**
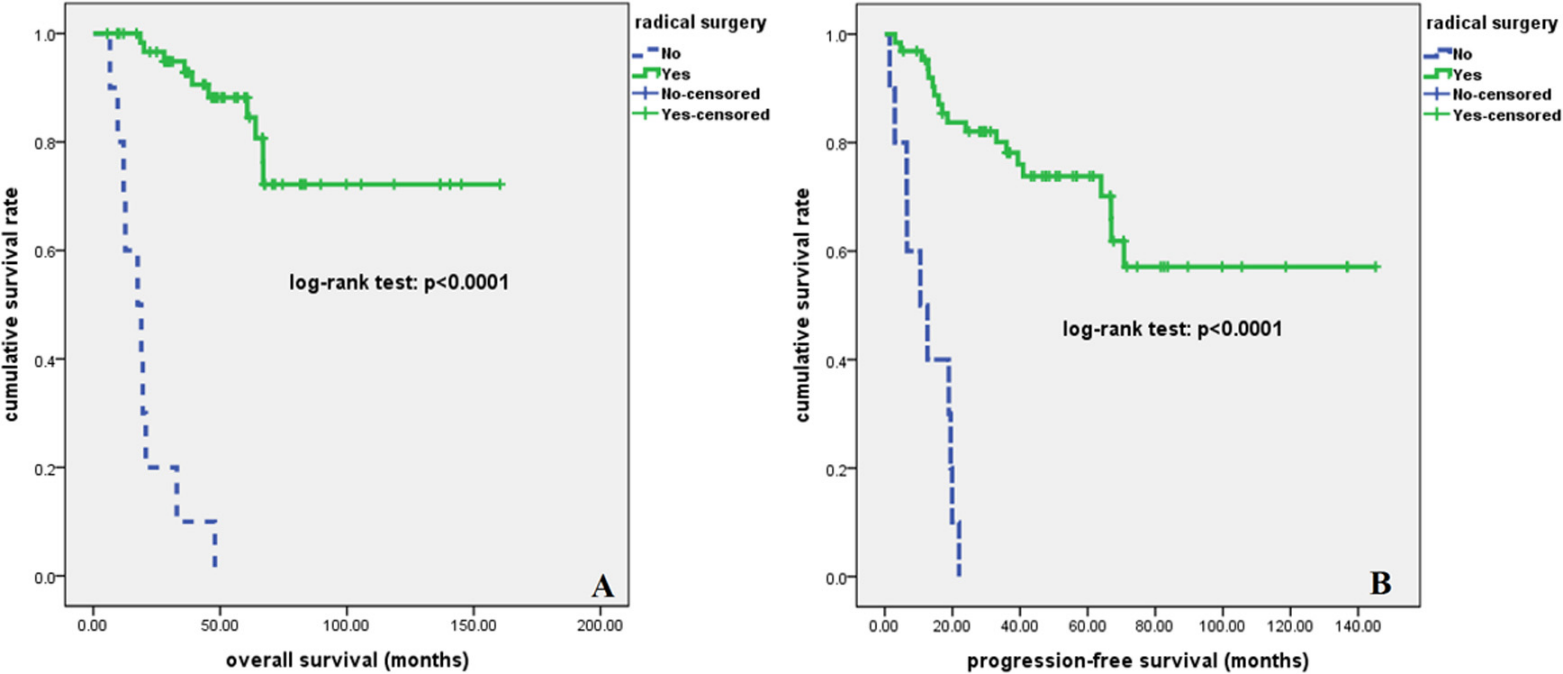
Survival curves according to treatment for all patients with pulmonary LELC. Patients who received radical surgery had significantly better OS (A) and PFS (B) than those who did not undergo radical surgery (both p<0.0001).

**Fig 4:**
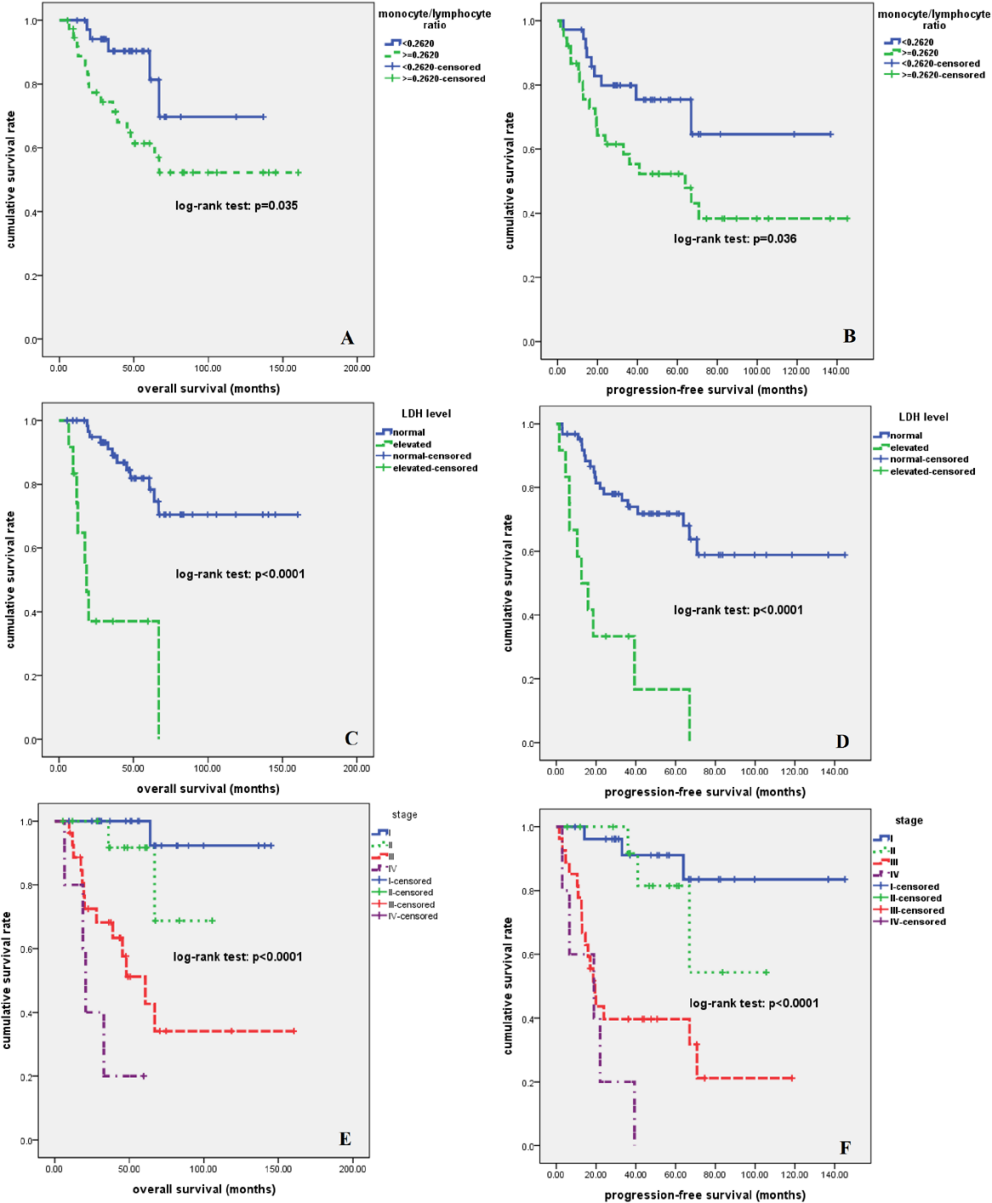
Kaplan-Meier survival analysis for all patients with pulmonary LELC. Higher MLR (A, B), elevated LDH level (C, D), and advanced stage (E, F) were significantly associated with inferior OS and PFS (p<0.05).

**Fig 5:**
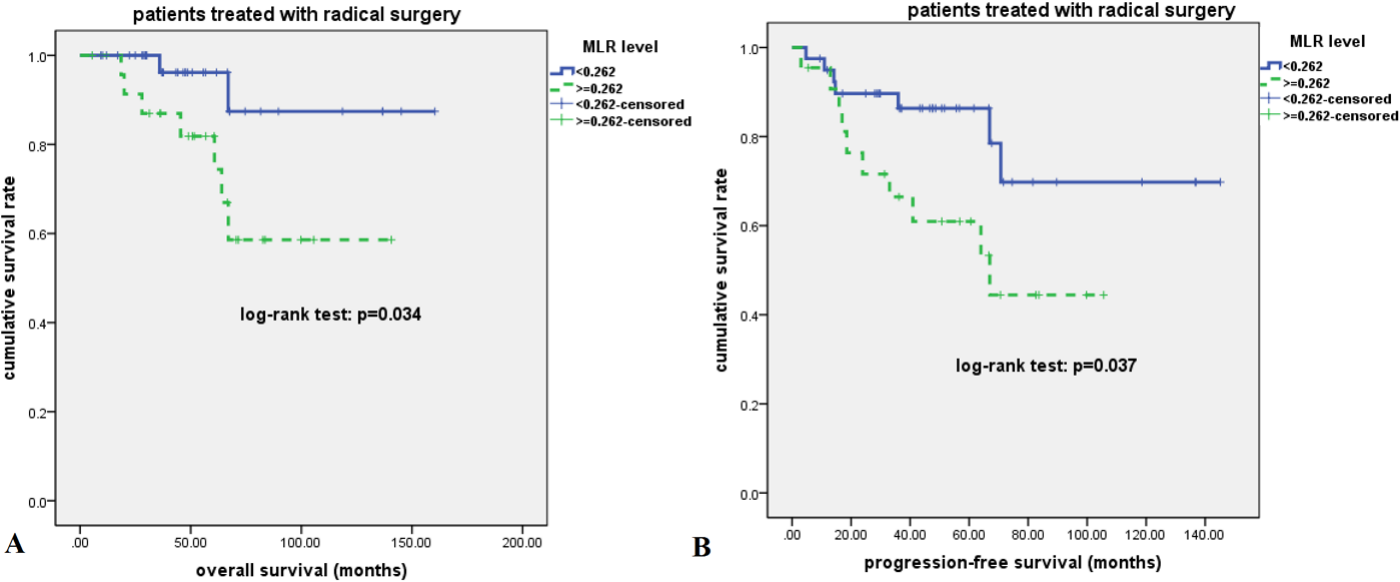
Kaplan-Meier survival analysis for patients of pulmonary LELC who received radical surgery. Higher MLR was significantly associated with inferior OS (A) and PFS (B) (p<0.05).

At a median follow-up time of 46 months (range, 5.5–160.4 months), 29 patients had disease relapse or progression at a median of 17 months (range, 1.5–70.8 months)., Twenty patients died of tumor at a median of 24 months (range, 6.6–67.0 months). The 2-year, 5-year, and 10-year OS rate were 86%, 72%, and 61%, respectively; the 2-year, 5-year, and 10-year PFS rate were 71%, 63%, and 49%, respectively **(Fig 2)**. As is shown in **Fig 3**, patients who received radical surgery had significantly better OS and PFS than those who did not have their tumor radically resected (both p<0.0001). In univariate analysis, advanced stage, elevated lactate dehydrogenase (LDH) level, and higher MLR value (> = 0.262) were significantly associated with poor OS and PFS (p<0.05, **Fig 4**). There was no significant correlation between survival outcomes (both PFS and OS) and age, gender, smoking history, tumor size, pretreatment anemia status, and serum albumin level (p>0.05). As is shown in Table 2, in a multivariate Cox regression model that included stage, LDH and MLR level, all of these three factors were found to be independent prognostic factors for both PFS and OS (p<0.05). According to the treatment strategy, patients who received radical surgery had significantly better OS and PFS than those without radical surgery (p<0.05, data not shown). As is demonstrated in **Fig 5**, in patients who received radical surgery, MLR level was significantly correlated with OS and PFS (p = 0.034 and 0.037, respectively). In those 10 patients who only received palliative chemotherapy, MLR level was significantly associated with OS (p = 0.009) but not PFS (p = 0.119).

**Table 2:** Multivariate analysis of prognostic factors for OS and PFS.

Parameters	OS			PFS		
	HR	95% CI	P value	HR	95% CI	P value
Stage	3.850	1.841–8.049	<0.0001	2.736	1.697–4.409	<0.0001
LDH level	3.000	1.154–7.798	0.024	2.411	1.050–5.537	0.038
MLR level	3.707	1.240–11.081	0.019	2.343	1.045–5.255	0.039

Abbreviations: OS, overall survival; PFS, progression free survival; HR, hazard ratio; CI, confidence interval; LDH, lactate dehydrogenase; MLR, monocyte to lymphocyte ratio.

## Discussion

In this large retrospective cohort study on patients with pulmonary LELC, we demonstrated that MLR level was an independent prognostic factor for both PFS and OS, and the prognostic value of MLR level retained in patients who received radical surgery, indicating the necessity of exploration of new treatment strategy for patients with inferior survival outcomes.

Primary pulmonary LELC accounts for about 0.92% of lung cancers [12], and less than 300 cases have been reported to date. In this study, only 21.6% of patients had smoking history (either current smokers or ex-smokers), similar to previous reports [13], indicating cigarette smoking may be not an etiology for pulmonary LELC [4]. Most of our patients had resectable disease, and many of them had no symptoms at diagnosis, which may contribute to the better prognosis of this entity.

Recently there is increasing evidence that the systemic immune response of the host is an important prognostic factor for cancer patients [14]. As previously reported [4,13], pulmonary LELC is histopathologically featured by nests of epithelial tumor cells separated by abundant lymphocytes. Since Begin et al [1] first reported pulmonary LELC in 1987, the association of EBV infection with pulmonary LELC has been observed. The presence of EBV was indicated by in situ hybridization for EBERs in all of our 74 patients. Natural history of malignancies linked with viral infections, which are frequently associated with chronic inflammation, is often influenced by the immunologic state of the host. High lymphocyte infiltration in virus-positive tumors may reflect both enhanced antigenicity associated with viral infection and virus-independent factors, such as E-selectin expression on vascular endothelium that promotes lymphocyte egress [15,16]. In certain types of cancer, tumor infiltrating lymphocytes (TILs) have been shown to be associated with improved prognosis [17,18]. Pulmonary LELC is characterized by intensive lymphocytic infiltrate, which may demonstrate a preexisting tumor inflammatory environment as a consequence of EBV triggering TILs, and this may explain its better outcome. Moreover, tumor-associated macrophages (TAMs), an important component of inflammatory infiltrating leukocytes which are supposed to be derived from the monocytes [19], may interact with tumor cells to promote tumor development by producing various cytokines and chemokines. We also found significant expression of TAMs in pulmonary LELC tissue and correlated with worse survival outcomes (data not published yet). Thus, both monocytes and lymphocytes play major roles in the inflammatory response of pulmonary LELC. Our study demonstrated that pretreatment MLR level was an independent prognostic factor for both PFS and OS in pulmonary LELC. Although the mechanisms for this correlation between MLR and survival have not been investigated, previous studies have found that each of these two leukocyte subsets or combination of MLR has been found to be independently associated with the prognosis of various cancers [6–10,14,20]. High number of monocytes in circulating blood may promote the potential creation of TAMs in tumor microenvironment [21], therefore contribute to the tumor progression or metastasis. In contrary, lymphocytes may play important roles in host immunity against tumor, thus help to control the tumor growth and eradicate the residual disease [14]. In all, the combined MLR derived from above variables will enlarge the prognostic value of each factor.

In consistent with previous reports [3,4,12,13], patients with pulmonary LELC fare better than patients with common types of NSCLC. In our study, the 2-year and 5-year OS rates were 86% and 72%, respectively, and there seems to be a survival plateau in our cohort, indicating some patients of pulmonary LELC could be cured using multi-modality treatments. As is shown above, most of pulmonary LELC patients had early stage or locally advanced disease, thus radical surgery is the mainstay treatment for these patients. However, patients with higher MLR had significantly higher relapse rate (5-year relapse rate: 40% vs. 14%, p<0.05) even though they received radical surgery, suggesting close monitoring of disease relapse should be applied in those patients with higher MLR, and multi-modality treatment strategies should be considered for adjuvant conditions.

In conclusion, this is the first report to demonstrate the independent prognostic role of MLR in patients with pulmonary LELC, and might guide us to optimize the treatment strategies. However, due to the relatively rarity of this disease and the limitation of a retrospective study, further studies performed in a multicenter or prospective manner are necessary to validate the prognostic value of MLR in pulmonary LELC.
